# Reverse-transcription, loop-mediated isothermal amplification assay for the sensitive and rapid detection of H10 subtype avian influenza viruses

**DOI:** 10.1186/s12985-015-0378-1

**Published:** 2015-09-17

**Authors:** Sisi Luo, Zhixun Xie, Liji Xie, Jiabo Liu, Zhiqin Xie, Xianwen Deng, Li Huang, Jiaoling Huang, Tingting Zeng, Mazhar I. Khan

**Affiliations:** Guangxi Key Laboratory of Animal Vaccines and New Technology, Guangxi Veterinary Research Institute, Nanning, Guangxi 530001 P.R. China; Department of Pathobiology and Veterinary Science, University of Connecticut, 61 North Eagleville Road Storrs, Connecticut, 06269-3089 USA

## Abstract

**Background:**

The H10 subtype avian influenza viruses (H10N4, H10N5 and H10N7) have been reported to cause disease in mammals, and the first human case of H10N8 subtype avian influenza virus was reported in 2013. Recently, H10 subtype avian influenza viruses (AIVs) have been followed more closely, but routine diagnostic tests are tedious, less sensitive and time consuming, rapid molecular detection assays for H10 AIVs are not available.

**Methods:**

Based on conserved sequences within the HA gene of the H10 subtype AIVs, specific primer sets of H10 subtype of AIVs were designed and assay reaction conditions were optimized. A reverse-transcription loop-mediated isothermal amplification (RT-LAMP) assay was established for the rapid detection of H10 subtype AIVs. The specificity was validated using multiple subtypes of AIVs and other avian respiratory pathogens, and the limit of detection (LOD) was tested using concentration gradient of *in vitro*-transcribed RNA.

**Results:**

The established assay was performed in a water bath at 63 °C for 40 min, and the amplification result was visualized directly as well as under daylight reflections. The H10-RT-LAMP assay can specifically amplify H10 subtype AIVs and has no cross-reactivity with other subtypes AIVs or avian pathogens. The LOD of the H10-RT-LAMP assay was 10 copies per μL of *in vitro*-transcribed RNA.

**Conclusions:**

The RT-LAMP method reported here is demonstrated to be a potentially valuable means for the detection of H10 subtype AIV and rapid clinical diagnosis, being fast, simple, and low in cost. Consequently, it will be a very useful screening assay for the surveillance of H10 subtype AIVs in underequipped laboratories as well as in field conditions.

## Introduction

The influenza A viruses belong to the family Orthomyxoviridae and are classified into 18 hemagglutinin (HA) and 11 neuraminidase (NA) subtypes based on their antigenic properties [1, 2]. Avian influenza viruses (AIVs) can be divided into two distinct groups based on their virulence: highly pathogenic avian influenza viruses (HPAIVs) and low pathogenic avian influenza viruses (LPAIVs). HPAIVs generally cause high morbidity and mortality in poultry flock, even directly infect human or cause death such as H5N1 in Hong Kong in 1997 [3], but the H7N9 of HPAIVs broke out and infected human was low pathogenic in poultry in 2013 [4]; LPAIVs such as H9N2 cause mild or no symptoms in poultry, wild birds and human, and most AIV subtypes are LPAIVs [5, 6].

Most H10 subtype AIVs belong to LPAIVs and have been brought into sharp focus since the winter of 2013. H10N8 was reported to cause human disease for the first time in December 2013 following the emergence of the H7N9 and H5N1 subtype AIVs that cause serious disease in humans and have become a threat to public health [7, 8]. As of 15 February 2014, three cases of human infection with A (H10N8) virus have been confirmed in Jiangxi Province, China, of whom two died [9]. H10N8 had been reported only two cases that water samples from the Dongting Lake in 2007 and a duck from Guangdong in 2012 in China before human cases [10, 11]. Currently, H10 subtype AIVs are generally well-known as human infection and followed more focus. Indeed, H10 subtype AIVs have been reported previously, and some of these viruses can cause disease in mammals. H10N4 was confirmed to cause interstitial pneumonia in minks [12], and H10N5 has been found in pigs [13]. H10N7 led to increased mortality and decreased egg production in chickens on a commercial poultry farm in Australia [14, 15]. Rapid detection is very important for the prevention and control of AIVs. Methods for the detection of the H5, H7 and H9 subtypes of AIVs have been developed and applied to surveillance and clinical care [16]. Currently, rapid assays for the detection and surveillance of H10 subtype AIVs are not available, and the development of an effective and applicable assay is urgently needed.

The loop-mediated isothermal amplification (LAMP) technique was invented in 2000 [17] and provides a powerful gene amplification tool with simple conditions requiring only a water bath at 58–65 °C and a 30–60 min incubation. LAMP amplification relies on the Bst DNA polymerase with highly auto-cycling strand displacement DNA synthesis activity. RT-LAMP assay is being used widely for the detection of AIVs such as H1, H3 and H9 subtypes and various viral pathogens [18–23]. In recent years, improvements in the LAMP assay, such as visual detection of amplified product by adding calcein or hydroxynaphthol blue (HNB) [24], which only require a temperature-controlled water bath and effectively avoid nucleic acid production pollution, have made it easier to apply in primary clinical setting or for field use. In this study, we developed an effective RT-LAMP assay with calcein and MnCl_2_ [25], to visual detect the H10 subtype AIVs, which might be suitable in the surveillance of the H10 subtype of AIVs for the rapid detection of H10 subtype AIVs in poultry and wild birds.

## Materials and methods

### Design of primers

The HA gene sequences of the H10 subtype AIVs were available downloaded from the NCBI Influenza Virus Resource Database and were compared and analyzed using the DNAstar software to identify conserved regions. Several primer sets were designed within the conserved regions using the LAMP primer design software (Primer Explorer V4 http://primerexplorer.jp/elamp4.0.0/index.html) for used in the H10-RT-LAMP assays. The specificity of the primer sets was evaluated using NCBI BLAST to confirm that no cross-reactivity with sequences from other avian pathogens would occur. Finally, an optimal set of primers was chosen after many rounds of analysis to ensure the highest specificity and sensitivity of the H10-RT-LAMP assay. The RT-LAMP primer set consists of two outer primers (F3 and B3), two inner primers (FIP and BIP, FIP = F1c + F2, BIP = B1c + B2), and two loop primers (LF and LB). The high-performance liquid chromatography (HPLC)-purified primer information is shown in Table 1.

**Table 1:** Primer information

Name	Primer sequences (5′-3′)	Genome position
FIP = F1c + F2	CATTGTGTGAGAAGGTGATATTA	F1c, 794-772
	-ACAATTTTGTTCCGGTTGTTG	F2, 681-701
BIP = B1c + B2	GATAGCACCGAGYCGAGTTAG	B1c, 802-822
	-ACAATTATTGTCTATTGGTGCAT	B2, 880-858
F3	TGGGACACAATCAYTGTCCA	634-653
B3	TATAGAACCCCCTCTCCAAA	913-894
LF	GAAAATCAATCCGCCCACTT	746-727
LB	ATTGGAAGAGGATTGGGGAT	830-849

Genome position according to the HA gene of A/duck/Guangdong/E1/2012(H10N8) (GenBank accession no JQ924786.1)

### Viral strains and DNA/RNA extraction

The reference and isolate strains or inactive strains (H5, H7, H14, H15 and H16) of AIV, human influenza virus (H1N1, H3N2, and Influenza B viruses), other avian pathogens and clinical samples used in this study are listed in Tables 2 and 3. The genomic DNA/RNA was extracted from 200 μL samples using a DNA/RNA Miniprep kit (Takara, Dalian, China) according to the protocol suggested by the manufacturer. The DNA and RNA were eluted with 40 μL of elution buffer and immediately stored at −80 °C until use.

**Table 2:** Sources of pathogens analyzed and H10-RT-LAMP assay results

Number	Virus strain	Source	Titre	H10-RT-LAMP	GenBank accession or laboratory references
1	A/duck/Guangxi/030D/2009 (H1N1)	GVRI	10^5.5^EID_50_/mL	-	KC608160
2	A/duck/Guangxi/N42/2009 (H3N2)	GVRI	10^5.4^EID_50_/mL	-	JN003630
3	A/duck/Guangxi/GXd-4/2009 (H6N6)	GVRI	10^6.8^EID_50_/mL	-	JX304746.1
4	A/turkey/Ontario/6118/1967 (H8N4)	UCONN	10^6.3^EID_50_/mL	-	GU053171.1
5	A/duck/Guangxi/RX/2009 (H9N2)	GVRI	10^6.9^EID_50_/mL	-	KF768236.1
6	A/duck/Guangxi/LAD9/2009 (H9N8)	GVRI	10^7.1^EID_50_/mL	-	KF768214.1
7	A/Turkey/MN/3/1979 (H10N7)	UCONN	10^6.7^EID_50_/mL	+	GU186627.2
8	A/duck/Alberta/60/1976 (H12N5)	UCONN	10^6.3^EID_50_/mL	-	AB288334
9	A/gull/Maryland/704/1977 (H13N6)	UCONN	10^6.5^EID_50_/mL	-	D90308.1
10	A/mallard duck/Astrakhan/263/1982 (H14N5)	UCONN	10^6.2^EID_50_/mL	-	CY014604.1
11	A/wedge-tailed shearwater/Western Australia/2576/1979 (H15N9)	UCONN	10^5.4^EID_50_/mL	-	CY006010.1
12	A/shorebird/Delaware/168/2006(H16N3)	UCONN	10^6.0^EID_50_/mL	-	EU030976.1
13	Newcastle disease virus (NDV): Lasota	GVRI	10^8.0^EID_50_/mL	-	JF950510.1
14	Infectious bronchitis virus (IBV): M41	GVRI	10^8.3^EID_50_/mL	-	DQ834384
15	Infectious laryngotracheitis virus (ILTV)	GVRI	10^5.8^EID_50_/mL	-	NC_006623.1
16	A/Mallard/Alberta/77 (H2N3)	UCONN	10^6.0^EID_50_/mL	-	Peng Y et al. (2011) [19]
17	A/Turkey/GA/209092/02 (H5N2)	UCONN	10^7.8^EID_50_/mL	-	Peng Y et al. (2011) [19]
18	A/Turkey/CA/35621/84 (H5N3)	UCONN	10^6.5^EID_50_/mL	-	Peng Y et al. (2011) [19]
19	A/waterfowl/GA/269452-56/03 (H5N7)	UCONN	10^7.0^EID_50_/mL	-	Peng Y et al. (2011) [19]
20	A/Turkey/WI/68 (H5N9)	UCONN	10^7.2^EID_50_/mL	-	Peng Y et al. (2011) [19]
21	A/Chicken/NY/273874/03 (H7N2)	UCONN	10^7.0^EID_50_/mL	-	Peng Y et al. (2011) [19]
22	A/Duck/HK/876/80 (H10N3)	HKU	10^6.0^EID_50_/mL	+	Xie Z et al. (2006) [16]
23	A/Chicken/Guangxi/90C/2011 (H1N2)	GVRI	10^6.3^EID_50_/mL	-	HA and HI test
24	A/Duck/Guangxi/070D/2010 (H4N6)	GVRI	10^6.2^EID_50_/mL	-	HA and HI test
25	A/chicken PA/3979/97 (H7N2)	PU	10^6.6^EID_50_/mL	-	HA and HI test
26	A/duck/PA/2099/12 (H11N9)	PU	10^6.0^EID_50_/mL	-	HA and HI test
27	A/Guangxi/1415/15 (H1N1)	GXCDC	10^5.8^EID_50_/mL	-	HA and HI test
28	A/Guangxi/1241/14 (H1N1)	GXCDC	10^6.0^EID_50_/mL	-	HA and HI test
29	A/Guangxi/1420 /15 (H3N2)	GXCDC	10^5.6^EID_50_/mL	-	HA and HI test
30	A/Guangxi/1632/15 (H3N2)	GXCDC	10^6.2^EID_50_/mL	-	HA and HI test
31	B/Guangxi/1418/15	GXCDC	10^5.8^EID_50_/mL	-	HA and HI test
32	B/Guangxi/1470/15	GXCDC	10^5.6^EID_50_/mL	-	HA and HI test

*GVRI* Guangxi Veterinary Research Institute, Nanning, Guangxi, China; *UCONN* University of Connecticut, USA; *PU* University of Pennsylvania, USA; *HKU* University of Hong Kong, China; *GXCDC* Guangxi Provincial Center for Disease Control and Prevention, China

**Table 3:** Results of two assays for the detection of H10 subtype AIVs in clinical samples

Sample type	Total samples	Number of positive samples	
		LAMP	Viral isolation
Chicken	198	0	0
Duck	611	4	4
Geese	234	2	2
Francolin	134	1	1
Pigeon	119	1	1
Total	1296	8	8

### RT-LAMP reaction

The H10-RT-LAMP assay was performed in a 25 μL reaction containing 2.5 μL 10 × Thermopol Reaction Buffer (New England Biolabs, Beijing, China), 8 U Bst DNA polymerase (large fragment; New England Biolabs, Beijing, China), 200 U reverse transcriptase M-MLV (Takara, Dalian, China), primers (for FIP and BIP, 1.6 μM each, for F3 and B3, 0.2 μM each, and for LF and LB, 0.8 μM of each), and 2 μL of the template RNA. Based on previous studies [18–23], the final concentration ranges of the three primary reagents, dNTPs (Takara, Dalian, China) (0.2 to 1.6 mM), betaine (Sigma-Aldrich) (0.2 to 1.4 M), and MgSO_4_ (Sigma-Aldrich) (2 to 9 mM), were optimized for amplification efficiency. The amplification reaction was tested at 58 to 65 °C for 30 to 60 min to find the optimal temperature and incubation time. Finally, the reaction was terminated by heating at 80 °C for 5 min. All the experiments were repeated three times.

Real-time monitoring of turbidity at the desired temperature was achieved by using an LA-320CE turbidimeter (Eiken Chemical Co. Ltd. Tochigi, Japan) that recorded the optical density (OD) at 650 nm every 6 s. Turbidity values greater than 0.1 were considered positive. In addition to the turbidity meter, reaction results could be observed visually based on a color changes of green for positive and orange for negative results by using 25 μM calcein (Eiken Chemical, Tochigi, Japan) combined with 0.5 mM MnCl_2_ (Eiken Chemical, Tochigi, Japan).

### *In vitro* transcription

Following the extraction of RNA from the A/duck/HK/876/80 (H10N3) strain, the HA gene was amplified by RT-PCR using previously reported primers [26]. The purified PCR product was cloned into the pGEM T-easy vector and transformed into *Escherichia coli* DH5α cells. The plasmid, named H10-pGEM, was sequenced and linearized using the restriction endonuclease *Spe* I. The plasmid was then used as a template for the RiboMax T7 (Promega, Madison, WI) *in vitro* transcription system according to the manufacturer’s instructions. After DNase treatment to eliminate residual DNA, the RNA product was purified using a RNA purification kit (Tiangen, Beijing), and its concentration was determined spectrophotometrically using a NanoDrop 2000 (Thermo Scientific, USA). The *in vitro* transcribed RNA was sequenced to further evaluate the direction of insert and the length. The right RNA transcribed from the HA gene from the H10 subtype AIVs was 10-fold serially diluted from 10^4^copies/μL to 1copy/μL as standard quantitatively.

### Specificity and sensitivity of the assay

To evaluate the specificity of the primer set used for the H10-RT-LAMP assay, DNA/RNA extracted from AIV subtypes H1, H2, H3, H4, H5, H6, H7, H8, H9, H10, H11, H12, H13, H14, H15, H16, human influenza viruses (H1N1, H3N2 and influenza B viruses), Newcastle disease virus (NDV), infectious bronchitis virus (IBV), and infectious laryngotracheitis virus (ILTV) were analyzed using the H10-RT-LAMP assay.

A 10-fold dilution series vitro-transcribed RNA ranging from 10^4^ copies/μL to 1copy/μL served as the standard to test the sensitivity of this assay. All reactions were performed in triplicate.

### Clinical sample detection

This study was approved, and the protocol sample collection was conducted by the Animal Ethics Committee of the Guangxi Veterinary Research Institute, which supervises all live bird markets (LBMs) in Guangxi province. Oral and cloacal swab samples were gently collected with permission from the owners of LBMs, and the fowls were not anesthetized before sampling and were observed for 30 min after sampling before being returned to their cages. The clinical samples were prepared in a viral transport medium, which was made up of 0.05 M phosphate buffered saline (PBS) containing antibiotics of penicillin (10,000 units/ml), streptomycin (10 mg/ml), gentamycin (10 mg/ml), kanamycin (10 mg/ml) and 5 % (*v/v*) fetal bovine serum, and were placed in the ice box.

A total of 1296 clinical swab samples (Table 3) including healthy chickens, duck, geese, francolins and pigeons were collected from five LBMs named Xiuxiang (No.1-246), Beihu (No.247-525), Sulu (No.526-813), Danchun ((No.814-1102) and Jiangqiao (No.1103-1296) in Nanning in Guangxi province and assayed by RT-LAMP and virus isolation. Virus isolations were prepared by inoculating specific-pathogen-free (SPF) embryonated chicken eggs and were tested using a hemagglutination assay (HA) and a hemagglutination inhibition (HI) assay described previously [27].

## Results

### Optimization of H10-RT-LAMP

Following standardization and optimization, the optimal of final concentrations were 1.4 mM each dNTP, 0.8 M betaine, 6 mM MgSO_4_. The H10-RT-LAMP reactions were incubated at 63 °C for 40 min and then heat-inactivated at 80 °C for 5 min to terminate the reaction.

### Specificity and sensitivity of the H10-RT-LAMP assay

**Fig. 1:**
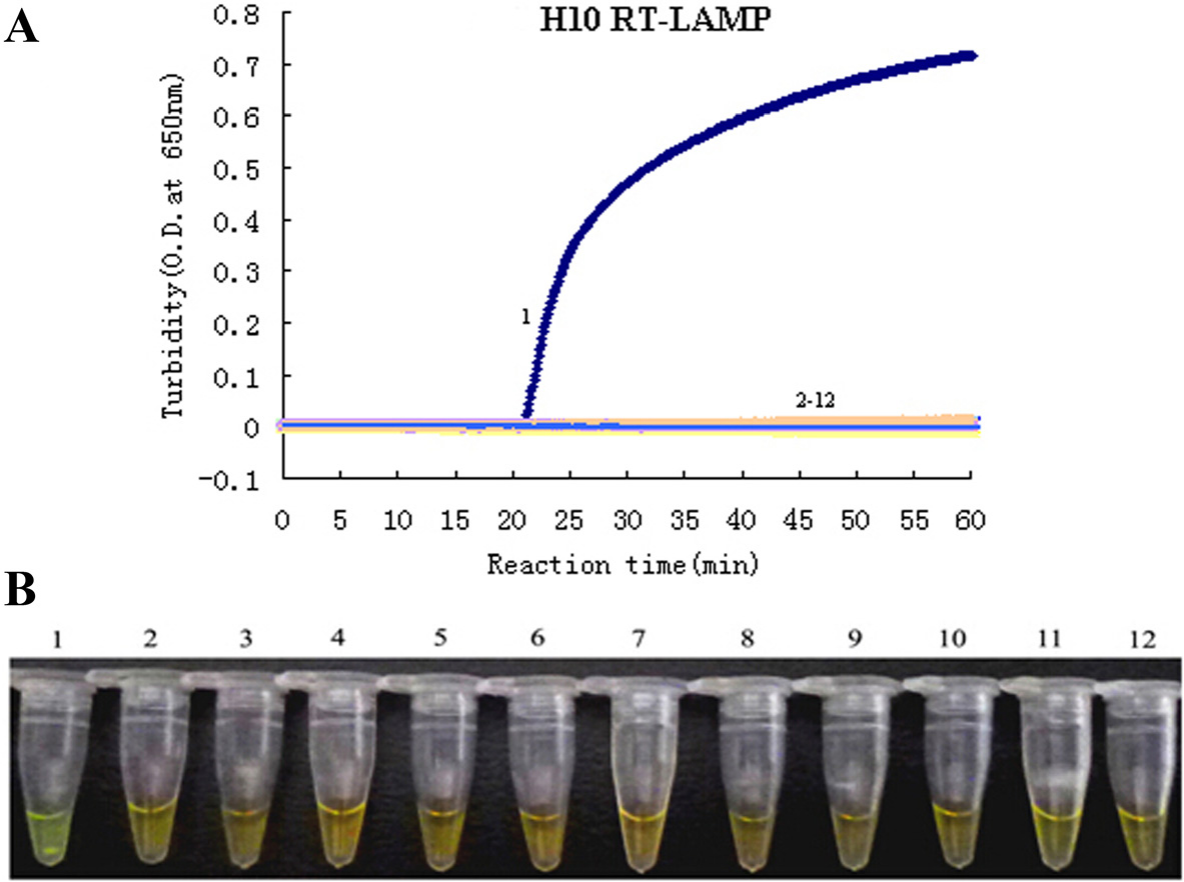
Specificity results of the H10-RT-LAMP assay. **a** The turbidity curve of the RT-LAMP products. **b** Visualization of the RT-LAMP products. 1, H10N3; 2, H1N1; 3, H3N2; 4, H5N3; 5, H6N8; 6, H7N2; 7, H9N2; 8, H11N9; 9, NDV; 10, IBV; 11, ILTV; 12, blank control

**Fig. 2:**
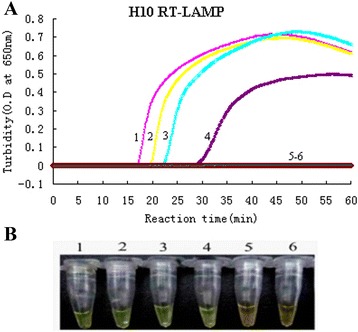
Sensitivity results of the H10-RT-LAMP assay. **a** The turbidity curves of the RT-LAMP products from a 10-fold dilution series of RNA. **b** Visualization of the RT-LAMP products from a 10-fold dilution series of RNA. 1, 10^4^ copies/μL; 2, 10^3^ copies/μL; 3, 10^2^ copies/μL; 4, 10 copies/μL; 5, 1 copy/μL; 6, blank control

As expected, the turbidity value of the H10 subtype AIVs gradually increased at 20 min. We observed that the increased turbidity curve appeared only when H10 subtype AIVs was used as the template that indicated a positive result, while straight lines were observed for the other avian pathogens (H1-H9 AIV, H11-H16 AIV, human influenza viruses, NDV, IBV and ILTV) indicating a lack of amplification (Fig. 1a). A green color was observed for the H10 subtype AIVs, and the other reactions remained the same orange color as before the incubation (Fig. 1b). The results of the detection assay for each strain are shown in Table 2. All the results suggest that this newly developed RT-LAMP assay only amplifies H10 subtype AIVs and shows no cross-reactivity with other avian pathogens and human influenza viruses (H1N1, H3N2 and influenza B virus), thus validating the high specificity of this H10-RT-LAMP assay. The sensitivity of the test was established by dilution series vitro-transcribed RNA standard samples. The LOD for the RT-LAMP was 10 copies/μL (Fig. 2a and b). Direct visualization of the Calcein dye-aided color change not only determined amplification but also did not require expensive instruments, needing only a temperature-controlled water bath, and the calcein dye results were consistent with the turbidity data.

### Clinical sample detection

To evaluate the sensitivity of the H10-RT-LAMP assay in clinical practice, a total of 1296 oral and cloacal swab samples were collected and subjected to methods of detection in parallel: H10-RT-LAMP, and viral isolation. Of the 1296 oral and cloacal swab samples tested, 8 were confirmed to be positive for H10 subtype AIVs for both RT-LAMP and viral isolation (Table 3).

## Discussion

According to the World Organisation for Animal Health (OIE), HPAIVs includes two characteristic i) high pathogenic *in vivo* and ii) presence of multiple basic amino acids at the HA cleavage site. Significantly, there were two H10 subtype AIVs [A/turkey/England/384/79(H10N4) and A/mandarin duck/Singapore/805/F-72/7/93(H10N5)] that lacked multi-basic amino acids at the HA cleavage site, a typical marker for HPAIVs, but are highly virulent in chickens according to the test *in vivo*, and been reported as HPAIVs by both the OIE and the EU definitions [28, 27]. Some findings have suggested that the HA molecule of H10 subtypes may have an affinity for human cell receptors and may also help the virus to adapt to multiply in humans [29]. Therefore, similar to H7N9, the first sign of a poultry infection could be the emergence of clinical cases of H10N8 in humans, which would then lead to the identification of silent outbreaks in poultry [8]. Therefore, the development of a simple, rapid, visual and effective assay for H10 subtype AIVs diagnosis would significantly aid efforts to control this disease. We need to enhance surveys and collect samples monthly, quarterly and annually to monitor the H10 subtype AIVs.

Virus isolation is a gold standard and fundamental diagnostic method for the detection of AIVs, the results are accurate and reliable, but the procedure is time consuming labor intensive to inoculate embryonated chicken egg for 2–5 days and harvest allantoic fluid for HI identification requiring the positive serum of multiple subtypes AIVs. Other molecular biological diagnostic techniques, such as conventional reverse transcription polymerase chain reaction (RT-PCR) and quantitative real-time RT-PCR (qRT-PCR) have been not reported for H10 subtype AIVs.

We see several obvious advantages of the RT-LAMP assay: 1) The specificity and sensitivity are greatly enhanced by six primers recognizing eight independent target sequence regions, whereas conventional RT-PCR primers only recognize two independent regions. 2) The RT-LAMP assay only requires small amounts of cDNA to accomplish efficient amplification. Furthermore, reverse transcription can proceed at the same temperature, 58–67 °C, as the LAMP reaction. Therefore, the two reactions can run at the same time, making LAMP a one-step assay for RNA-based pathogens that require reverse transcription for molecular detection. 3) RT-LAMP is simple and easy to perform and only requires a temperature-controlled water bath, making it suitable for primary clinical settings or field use. Therefore, we believe that this simple, fast, and effective assay might be applied to the field detection of H10 subtype AIVs infections. The RT-LAMP assay has many advantages as mentioned above, there is still a problem to note. The RT-LAMP assay has great amplification efficiency so the reaction product can form aerosol to contaminate the surroundings when opening the tube. To avoid the contamination in this study, the fluorescence reagent (Calcein and MnCl_2_) were added to RT-LAMP reaction mixture before amplification in order to inspect the result of RT-LAMP assay directly with the naked eye.

In this study, we used calcein’s color change to visually determine results; we also monitored turbidity using the Loopamp Realtime Turbidimeter. To our knowledge, this is the first study to explore the use of H10 AIVs LAMP technology in a diagnostic test for H10 AIVs. This assay and its analysis are simple, specific, sensitive and rapid, do not require special equipment, and can be applied to detect H10 AIVs in clinical samples.

## Conclusions

In summary, this newly developed H10-RT-LAMP assay is a specific, sensitive, rapid, and cost-effective assay for the rapid detection and epidemiological surveillance of the H10 subtype AIVs. It can be applied to the rapid visual diagnosis of clinical samples and can also provide an effective tool to prevent and control H10 subtype AIVs disease.
